# *Ex vivo* cultures combined with *vivo*-morpholino induced gene knockdown provide a system to assess the role of WT1 and GATA4 during gonad differentiation

**DOI:** 10.1371/journal.pone.0176296

**Published:** 2017-04-20

**Authors:** Lucas J. Rudigier, Christof Dame, Holger Scholz, Karin M. Kirschner

**Affiliations:** 1 Institut für Vegetative Physiologie, Charité-Universitätsmedizin Berlin, Campus Charité Mitte, Berlin, Germany; 2 Klinik für Neonatologie, Charité-Universitätsmedizin Berlin, Campus Virchow-Klinikum, Berlin, Germany; University of Texas at Austin Dell Medical School, UNITED STATES

## Abstract

Gonad morphogenesis relies on the correct spatiotemporal expression of a number of genes that together fulfill the differentiation of the bipotential gonad into testes or ovaries. As such, the transcription factors WT1 and GATA4 are pivotal for proper gonadal development. Here we address the contributions of GATA4 and WT1 to the sex differentiation phase in testes and ovaries. We applied an *ex vivo* technique for cultivating gonads in hanging droplets of media that were supplemented with *vivo*-morpholinos to knockdown WT1 and GATA4 either alone or in combination at the same developmental stage. We show that WT1 is equally important for both, the initial establishment and the maintenance of the sex-specific gene expression signature in testes and ovaries. We further identified *Foxl2* as a novel putative downstream target gene of WT1. Moreover, knockdown of WT1 reduced mRNA levels of several molecular components of the hedgehog signaling pathway in XY gonads, whereas *Gata4 vivo*-morpholino treatment increased transcripts of *Dhh* and *Ptch1* in embryonic testes. The data suggest that for its proper function, WT1 relies on the correct expression of the GATA4 protein. Furthermore, GATA4 down-regulates several ovarian promoting genes in testes, such as *Ctnnb1*, *Fst*, and *Bmp2*, suggesting that this repression is required for maintaining the male phenotype. In conclusion, this study provides novel insights into the role of WT1 and GATA4 during the sex differentiation phase and represents an approach that can be applied to assess other proteins with as yet unknown functions during gonadal development.

## Introduction

The bipotential genital ridge is a unique tissue in the embryo because it can develop into either testes or ovaries. Cell fate decisions with respect to the male or female phenotype constitute a counterbalanced system, in which the activation of one pathway leads to the repression of the other one [1]. This bifurcation step is accomplished by a complex regulatory network, within which the two zinc finger transcription factors, WT1 (Wilms tumor 1) and GATA4 (GATA binding protein 4), play prominent roles [2].

Embryonic development requires the correct expression of WT1 and GATA4. Homozygous knock-out mice for *Wt1* and *Gata4* are embryonic lethal and exhibit several abnormalities including gonadal and myocardial malformations [3–5]. In addition, WT1 and GATA4 are essential for the initial development of the genital ridge [6,7]. Testis development occurs in the presence of *Sry* (sex determining region Y), and WT1 has been implicated in its regulation [8]. Remarkably, selective ablation of the WT1 protein isoform, that contains an additional three amino acids insertion, lysine–threonine–serine (KTS), in zinc finger three causes complete male-to-female sex reversal, most likely as a consequence of diminished SRY protein levels [9]. The regulation of the *Sry* gene, however, occurs synergistically with GATA4 and NR5A1/SF1 (nuclear receptor subfamily 5 group A member 1/ steroidogenic factor 1) [10]. Thus, it is not surprising that conditional deletion of *Gata4* by embryonic day 10.5, but not at later stages, culminates in a sex-reversed phenotype emphasizing the role of GATA4 during the sex determination phase [11]. In order to fulfill its normal function, GATA4 interacts with the co-factor FOG2 (friend-of-GATA). Indeed, mice carrying a *Gata4* knock-in allele, which abrogates the binding of GATA4 to FOG2 show abnormal differentiation of XY gonads [12].

Conditional gene targeting in mice revealed the essential roles of WT1 and GATA4 in gonadal development and provided important insights into the individual function of each transcription factor [9,12]. Yet, it is still unclear how WT1 and GATA4 interact during sex differentiation in testes and ovaries. This shortcoming is most likely due to the lack of an experimental model allowing for the simultaneous and controlled knockdown of both genes in the gonads at defined developmental stages.

Thus, additional experimental tools are needed to dissect the presumed interplay between WT1 and GATA4, particularly during the later phases of gonadal development. Ideally, these tools facilitate a rapid and precise analysis and as such provide the means to functionally explore the involvement of novel candidate genes [13], which have been previously identified and thus await *in vivo* validation [14–16]. Along these lines, shRNAs or morpholino oligomers have been successfully used to knockdown candidate genes [17,18]. While the injection of shRNAs into bipotential gonads caused a restricted sex reversal at the site of application, tissues are highly susceptible to mechanical damage due to the injection procedure [18]. In this situation, the transfection of *ex vivo* organ cultures with *vivo*-morpholinos may provide an alternative approach to silence one or several genes in developing testes and ovaries without causing tissue injury. *Vivo*-morpholinos have previously been applied to knockdown *Wt1* and other genes in kidney explants [19,20].

In the present study, we applied *vivo*-morpholinos to murine embryonic gonads grown *ex vivo* in hanging droplets of culture media. This experimental setting was used to examine the contributions of GATA4 and WT1 to the sex differentiation phase in testes and ovaries. To this end, we inhibited GATA4 and WT1 protein translation, either alone or in combination, and subsequently determined gene expression patterns and changes in gonadal cell proliferation.

The data show that this strategy allows, in a fast and reliable manner, not only to efficiently knockdown critical transcription factors in gonadal explants, but also to analyze downstream effects at the same developmental stage in testes and ovaries. Furthermore, the results indicate that WT1 is equally important for both, the initial establishment and the maintenance of a sex-specific gene expression signature. These effects of WT1 require normal levels of the GATA4 protein. Our data also demonstrate that GATA4 inhibits typical ovarian promoting genes, e.g. *Ctnnb1* (Catenin beta 1), *Fst* (Follistatin), *and Bmp2* (Bone morphogenetic protein 2), in the male gonads suggesting that this repression is required for maintaining the testis phenotype.

## Materials and methods

### Animals

All procedures were performed according to the Animal Protection Law guidelines and approved by the legal authorities represented by the Landesamt für Gesundheit und Soziales Berlin (LAGeSo). Experiments were conducted under permit no. T0308/12 issued by the LAGeSo. Heterozygous *Wt1*^*+/-*^ breeding pairs (C57BL6 strain, The Jackson Laboratory, Bar Harbor, ME) were mated in the local animal facility. The morning of vaginal plug was considered 0.5 days post conception (dpc). Timed pregnant mice were anesthetized with isoflurane and sacrificed by cervical dislocation. Embryos were collected and genotyped as previously described [21].

### Culture of embryonic gonads

Embryos were collected from timed pregnant mice (C57BL6 strain) and tail somites were counted to determine the embryonic stage [22]. Gonads with attached mesonephroi were dissected at the indicated developmental stages. Sex determination of the embryos was performed by PCR amplification of the Y chromosomal gene *Kdm5d* from genomic DNA using the following primers: mKdm5d-F, 5’-CTGAAGCTTTTGGCTTTGAG-3’; mKdm5d-R, 5’ CCACTGCCAAATTCTTTGG-3’ [23]. For pairwise comparison, the gonads of each embryo were cultivated separately in a hanging droplet of DMEM nutrient with stable L-glutamine (PAA Laboratories, Cölbe, Germany) supplemented with 10% FCS (Biochrom, Berlin, Germany), 100 IU/ml penicillin (PAA Laboratories) and 100 μg streptomycin (PAA Laboratories) according to a modified protocol [24]. The explants were grown in a humidified 5% CO_2_ chamber at 37°C and transfected with *vivo*-morpholino oligomers (Gene Tools, Philomath, OR) as described [20]. The organ cultures were processed after 72 h incubation in the presence of the following *vivo*-morpholinos used at 10 μM concentrations each for sequence-specific inhibition of protein translation:

5’-CAGGTCCCGCACGTCGGAACCCAT-3’ (*Wt1 vivo*-morpholino),
5’-CAGCTCCGGCACCTCGCAACCGATG-3’ (*Wt1* mismatch *vivo*-morpholino).
5’-GGCCATGGCCAGGCTTTGGTACATC-3’ (*Gata4 vivo*-morpholino),
5’-GGCGATGCCCAGCCTTTCGTAGATC-3’ (*Gata4* mismatch *vivo*-morpholino).

### BrdU labeling of cultured embryonic gonads

To identify proliferating cells, 5-bromo-2-deoxyuridine (BrdU, 10 **μ**M) was added to the culture medium 24 h before harvest. Organs were fixed in 10% (v/v) formalin (J.T. Baker, München, Germany) for 20 min, incubated in quenching solution (1x PBS/ 50mM NH_4_Cl) for 30 min and embedded into moulds covered with TissueTek^®^ (Sakura Finetek, Staufen, Germany). Embedded organs were stored at -80°C until cryosectioning. For immunostaining, 8 μm cryosections were heated in a microwave oven for 7 min in 1x citrate buffer (1.8 mM citric acid, 8.2 mM trisodium citrate, 0.05% (v/v) Tween 20, pH 6.0) and subsequently blocked for 10 min at room temperature in serum-free DakoCytomation protein block (Dako, Hamburg, Germany). To detect proliferating cells, a biotinylated antibody against BrdU (51-75512L, BD Pharmingen, Heidelberg, Germany) was used together with antibodies against WT1 (rabbit anti-WT1, N-180, Santa Cruz Biotechnology, Heidelberg, Germany) or GATA4 (goat anti-GATA4, C-20, Santa Cruz Biotechnology), each diluted 1:100 in blocking solution. Tissue sections were incubated with primary antibodies for 1 h at room temperature and then washed in 1x PBS/Tween20. Strepdavidin-Cy3 (Sigma-Aldrich, Munich, Germany) was used for visualization of the bound primary anti-BrdU antibody. The use of secondary antibodies for the detection of WT1 and GATA4 is described below. The cell nuclei were counterstained with Dapi (4’,6-diamino-2-phenylindole). Gonads were mounted using Vectashield^®^ mounting medium (H-1000, Vector Laboratories, Eching, Germany) and examined under an upright epifluorescence microscope (BX61, 20x objective, Olympus, Hamburg, Germany) equipped with a motorized stage (H1P1BX, serial no. 66404, Prior Scientific, Cambridge, UK). BrdU-positive cells were quantified using the macro feature in ImageJ. All images were post-processed in the same way. Briefly, RGB images were split in separate channels. A region of 1000 x 500 pixels was selected for analysis. The red (BrdU) and the blue (Dapi) channels were filtered using an unsharp mask-, medium- and maximum filter with a radius of 2 pixels. Background noise was subtracted, a threshold was set, and the cell area was measured. The BrdU-positive cell area was normalized to the total Dapi-positive area.

### Preparation of RNA, reverse transcription (RT) and quantitative PCR

Total RNA from single gonads was isolated using the RNeasy MicroKIT (Qiagen, Hilden, Germany). First strand cDNA synthesis was conducted with oligo(dT)12-18 primers and SuperscriptTM III reverse transcriptase (Life Technologies, Darmstadt, Germany). Quantitative real-time PCR (qRT-PCR) was performed in duplicates using a SYBR Green Master mix (Roche Diagnostics, Mannheim, Germany) in combination with the StepOnePlusTM system (Life Technologies) as described [25]. The sequences of the PCR primers are listed in Table 1. Relative expression levels were obtained by subtracting the Ct value of the housekeeping gene (Gapdh or Sdha) from the Ct value of the gene of interest. Differences in mRNA levels were calculated according to the equation 2^ΔΔCt^ as reported previously [26].

**Table 1:** qRT-PCR primers.

primer name	gene	NCBI no. (*mus musculus*)	sequence 5'-3'
Gata4-fwd	*Gata4*	NM_008092	GATGGGACGGGACACTACCTG
Gata4-rev			ACCTGCTGGCGTCTTAGATTT
Gapdh-fwd	*Gapdh*	NM_002046.5	ACGACCCCTTCATTGACCTCA
Gapdh-rev			TTTGGCTCCACCCTTCAAGTG
Wt1-fwd	*Wt1*	NM_144783	TGCCCTTCTGTCCATTTCACT
Wt1-rev			GATGTTCCCCAATGCGCCCTA
Dax1-fwd	*Dax1*	NM_007430.4	TGCTTGAGTTGGCCCAAGAT
Dax1-rev			AGGATCTGCTGGGTTCTCCA
Wnt4-fwd	*Wnt4*	NM_009523.2	GAGCAATTGGCTGTACCTGGC
Wnt4-rev			CCTGCTGAAGAGATGGCGTA
β-catenin-fwd	*β-catenin*	NM_007614.3	CGCCGCTTATAAATCGCTCC
β-catenin-rev			CAGGTCAGCTTGAGTAGCCA
Fst-fwd	*Fst*	NM_008046.2	AGTGACTTACTCCAGCGCCT
Fst-rev			CCGTTTCTTCCGAGATGGAGTT
Foxl2-fwd	*Foxl2*	NM_012020.2	AGCCGGCTTTTGTCATGATGG
Foxl2-rev			AGGTTGTGGCGGATGCTATT
Nr5a1-fwd	*(Nr5a1)Sf1*	NM_139051	CTGCCGCTTCCAGAAGTGCCT
Nr5a1-rev			GAGATGGGGCTCCAAAGTCAC
Sox9-fwd	*Sox9*	NM_011448.4	ACGCGGAGCTCAGCAAGACTC
Sox9-rev			GGTCGGCGGACCCTGAGATTG
Amh-fwd	*Amh*	NM_007445	GGGCCTGGCTAGGGGAGACTG
Amh-rev			CCCGCTGGGAAGTCCACGGTT
Amhr2-fwd	*Amhr2*	NM_144547.2	CGCTTTATCACTGCTGGC
Amhr2-rev			CTTCCCGAATGAGCACAT
Star-fwd	*StAR*	NM_011485	TGGATGGGTCAAGTTCGACG
Star-rev			CTCTGCAGGACCTTGATCTCC
Sdha-fwd	*Sdha*	NM_023281.1	ACCGGCTTGGAGCAAATTCT
Sdha-rev			TCCAAACCATTCCCCTGTCG
Dhh-fwd	*Dhh*	NM_007857.5	AGCAACTTGTGCCTCTGCTA
Dhh-rev			TGGAGTGAATCCTGTGCGTG
Ptch-fwd	*Ptch1*	NM_008975.2	TGGAGCAGATTTCCAAGGGGA
Ptch-rev			TTCTCGACTCACTCGTCCAC
Smo-fwd	*Smo*	NM_176996.4	CAAGCTCGTGCTCTGGTCC
Smo-rev			TTGTACCTCGTTTGGGCAGC
Gli1-fwd	*Gli1*	NM_010296.2	GGTGCACCACATCAACAGTG
Gli1-rev			GCTGCAACCTTCTTGCTCAC
Gli2-fwd	*Gli2*	NM_001081125.1	CACTCCAGCCAAGTTGGGAT
Gli2-rev			CTGCTGAGGAACTCCGTGG
Gli3-fwd	*Gli3*	NM_008130.2	TCTGAGTCCTCACAGAGCAAGC
Gli3-rev			ACAAGCTGATCTTGGGTGTCG

### SDS-PAGE and immunoblotting

Cells and tissues were lysed in Laemmli buffer and processed as described in detail elsewhere [27]. After separation on a denaturing 10% polyacrylamide gel, immunoblotting was performed consecutively with antibodies against WT1 (C19, Santa Cruz Biotechnology), GATA4 (C-20, Santa Cruz Biotechnology), and pan-Actin (clone C4, Millipore, Darmstadt, Germany). Peroxidase-coupled secondary antibodies (Santa-Cruz Biotechnology) were visualized with the Western Lightning Plus ECL reagent (PerkinElmer Life Sciences, Rodgau, Germany).

### Immunohistochemistry

Gonads were obtained from mouse embryos at the indicated developmental stages and fixed in 10% (v/v) formalin for 20 min. After incubation in quenching solution (1x PBS/ 50 mM NH_4_Cl) for 30 min, the whole-mount preparations were blocked overnight at 4°C in 1x PBS, 0.2% bovine serum albumin, 0.05% Triton X- 100. Double-immunofluorescent stainings were performed with antibodies against WT1 (monoclonal rabbit anti-WT1, ab89901, Abcam, Cambridge, UK) and GATA4 (goat anti-GATA4, C-20, Santa Cruz Biotechnology) diluted 1:100 in antibody diluent (Life Technologies) and applied simultaneously overnight at 4°C. Normal sera (Jackson ImmunoResearch, Hamburg, Germany) from the donor species were used as a negative control. For visualization of the bound primary antibodies Alexa Fluor 488-AffiniPure donkey anti-goat IgG (1:100 dilution, Jackson ImmunoResearch) and Cy3-AffiniPure donkey anti-rabbit IgG (1:200 dilution, Jackson ImmunoResearch, Suffolk, UK) were used. The cell nuclei were counterstained with bisbenzimide H33342 (Sigma-Aldrich). Overview images were acquired with an epifluorescence microscope (Axiovert100, Carl Zeiss, Jena, Germany) attached to a digital camera (Spot RT Slider, Diagnostic Instruments, Sterling Heights, MI). High resolution pictures were taken on a confocal microscope (Leica DM 2500, Leica Microsystems, Wetzlar, Germany) equipped with the LAS AF Lite software (Leica Microsystems). Image post-processing was performed with the ImageJ software and Adobe Photoshop.

### Statistics

Data analysis and visualization was performed using R [28] and the ggplot2 package [29]. Student’s *t*-test and ANOVA with Tukey’s post hoc test were performed as indicated. *P* values less than 0.05 were considered statistically significant.

## Results

### WT1 is essential for the establishment and maintenance of a sex-specific gene expression signature

**Fig 1:**
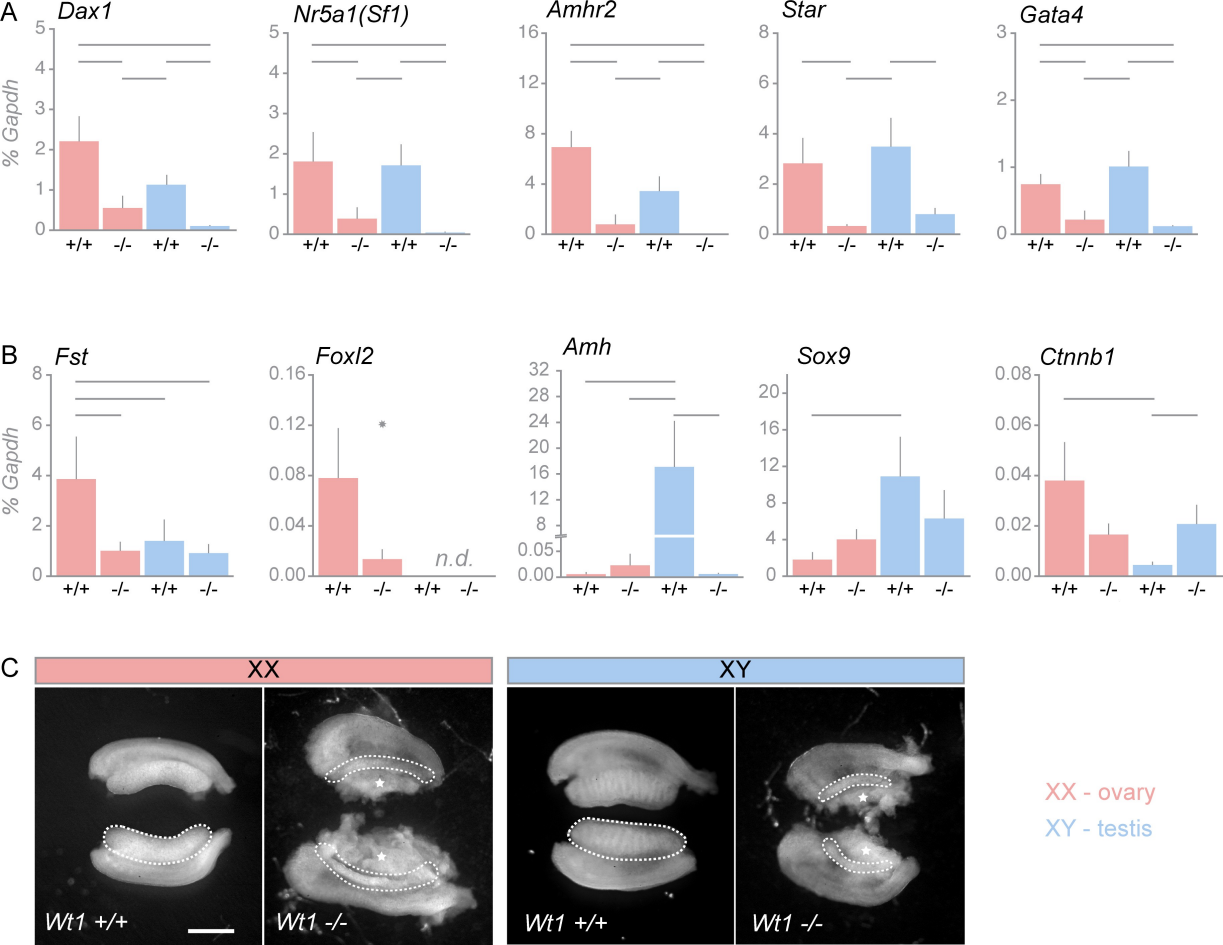
Deletion of *Wt1* results in the disruption of a sex-specific signature in XX and XY gonads. (A, B) Transcripts were measured by qRT-PCR in wild-type (*Wt1*^*+/+*^*)* and *Wt1*-deficient (*Wt1*^*-/-*^) XX and XY gonads. Genes are classified as being expressed at similar levels in both sexes or as exhibiting a clear predominance in either XX or XY gonads. Loss of *Wt1* showed a significant change in expression of (A) *Dax1*, *Nr5a1(Sf1)*, *Amhr2*, *Star*, and *Gata4* in both sexes. Expression of (B) *Fst*, *Foxl2*, *Amh2*, *Sox9*, and *Ctnnb1* was changed sex-specifically. (C) Representative morphology of *Wt1*^*+/+*^ and *Wt1*^*-/-*^ XX and XY gonads (marked by dashed lines) with attached mesonephroi. Tissues were obtained from embryos at 13.5 dpc. Note the disrupted morphology (dashed lines) in the *Wt1*^*-/-*^ XY and XX gonads. Scale bars indicate 500 μm. For qRT-PCR data (A, B) relative transcript levels were normalized to *Gapdh (*2^-ΔCt^*)* and shown in percent. Error bars indicate S.E.M. calculated from independent biological replicates (n ≥ 5). Statistical significances are marked by brackets (ANOVA with Tukey’s post hoc test) and asterisk (*p<0.05, *t*-test). n.d. = not detectable.

To gain further insight into the molecular mechanisms of gonadal development, we compared gene expression states in gonads derived from *Wt1*-deficient (*Wt1*^*-/-*^*)* murine embryos (13.5 dpc) to those of wild-type (*Wt1*^*+/+*^*)* littermates. This approach allows one to assess the long-term effect of WT1 depletion from the bipotential stage through sex determination until mid-gonadal morphogenesis. In order to analyze the role of WT1 in the implementation of a sex-specific gene expression signature, we distinguished between male and female sexes and analyzed a set of genes with known functions during ovarian development, e.g. *Foxl2* (forkhead box L2), *Fst* and *Ctnnb1*, and testis formation, e.g. *Nr5a1(Sf1)*, *Sox9* (SRY(sex determining region Y) -box 9), *Amh* (anti-Müllerian hormone), *Amhr2* (anti-Müllerian hormone receptor type 2) and *Star* (steroidogenic acute regulatory protein). In addition, the expression of *Nr0b1* (nuclear receptor subfamily 0 group B member 1, *Dax1*) and *Gata4*, both of which are involved in gonadal development of either sex, was studied. Two distinct expression patterns were discerned in *Wt1*^*-/-*^ gonads of 13.5 dpc staged embryos. Firstly, mRNAs such as *Dax1*, *Nr5a1*, *Amhr2*, *Star*, and *Gata4* were reduced in *Wt1*-deficient gonads independent of sex (Fig 1A). Secondly, genes with significantly different mRNA levels in wild-type XY and XX gonads, i.e. *Fst*, *Foxl2*, *Amh*, *Sox9*, and *Ctnnb1*, were down-regulated in a sex-specific manner (Fig 1B). *Fst* and *Foxl2* transcripts, which are essential during ovarian development, were significantly reduced in *Wt1*-deficient XX but not in XY gonads. Indeed, *Foxl2* transcripts were not detectable in embryonic XY gonads. *Amh*, which is required for regression of the Müllerian duct in males, was down-regulated in *Wt1*-deficient XY gonads, but barely detectable in ovaries of either wild-type or *Wt1*^-/-^ embryos (Fig 1B). *Sox9* mRNA levels were significantly higher in wild-type XY than in XX gonads, and this difference was lost in the absence of WT1. Lastly, *Ctnnb1* mRNA, which encodes for β-catenin and is crucial for female gonadogenesis was increased exclusively in *Wt1*^*-/-*^ XY gonads (Fig 1B). These results indicate that WT1 is essential for establishing a male- and female-specific gene expression signature during gonadal development. Furthermore, loss of this sex-specific gene expression pattern is associated with disrupted morphology of *Wt1*-deficient XY and XX gonads (Fig 1C). In *Wt1*^*-/-*^ XY gonads the seminiferous tubules could not be identified, limiting the macroscopic distinction between male and female murine gonads. Whilst *Wt1*-deficient gonads were also reduced in size, *Wt1*^*-/-*^ mesonephroi appeared to be rather enlarged in both sexes (Fig 1C). These data show that WT1 is required for establishing and maintaining a sex-specific gene expression signature in both, testes and ovaries and confirm its role in gonadal morphogenesis.

### WT1 and GATA4 are co-expressed during embryonic development in testes and ovaries

**Fig 2:**
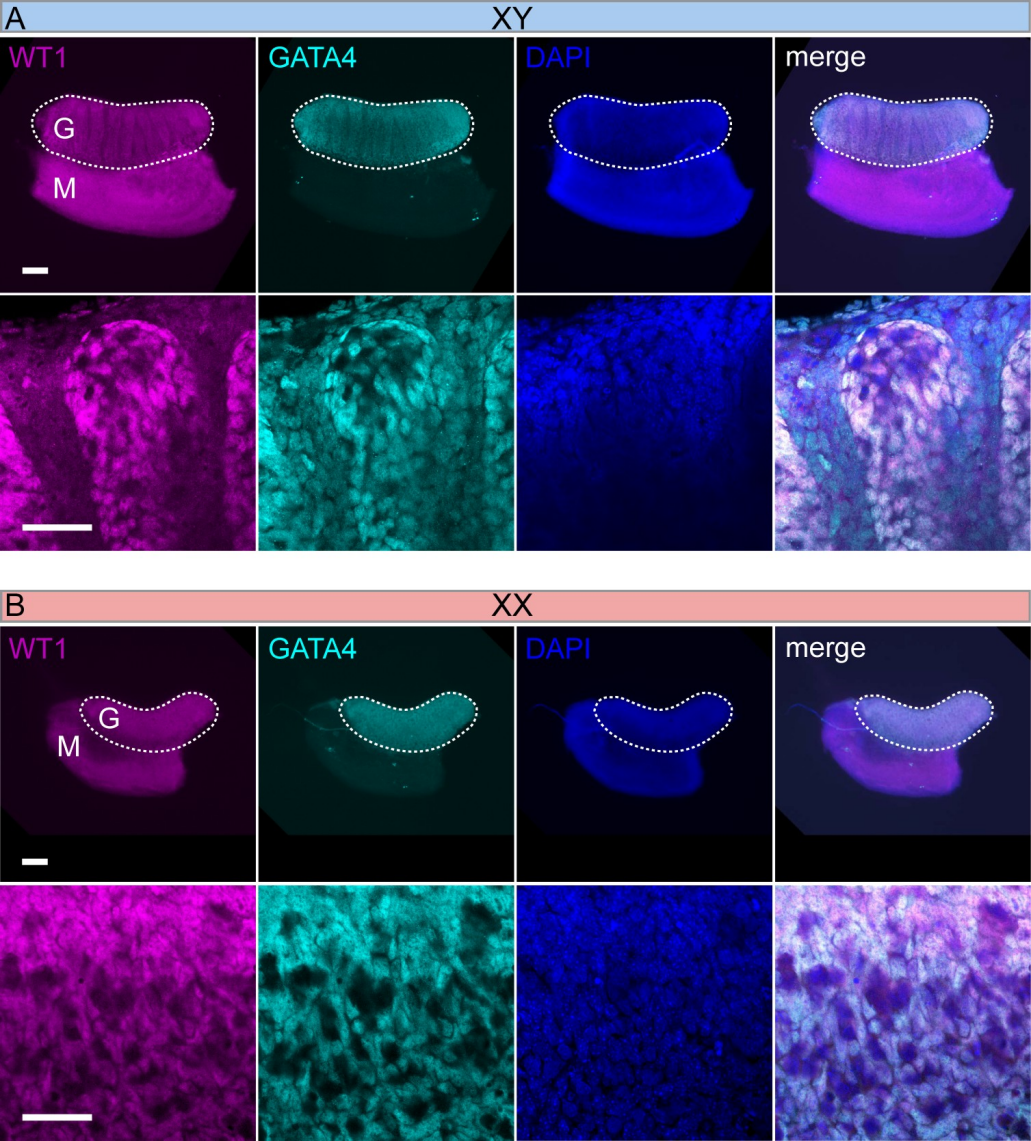
Co-expression of WT1 and GATA4 in XY and XX gonads. (A, B) Dissected XY and XX gonads (13.5 dpc) were co-immunostained as whole-mounts using antibodies against WT1 and GATA4. Bound primary antibodies were visualized with Cy3- (GATA4) and 488 dye (WT1) conjugates. Nuclear co- localization of WT1 and GATA4 is shown in the merged images. Cell nuclei were stained with Dapi. Scale bars are 200 μm (low-power magnification) and 100 μm (high-power magnification), respectively. G = gonad. M = mesonephros.

WT1 and GATA4 have prominent roles during gonad development in both sexes. Both proteins have been reported to act in a synergistic manner on the transcription of certain genes [30]. In order to address the spatial relationship between WT1 and GATA4, we examined whether both proteins are co-expressed in the developing gonads of wild-type mice. WT1, an established marker of Sertoli cells [31], was co-localized with GATA4 in these cells, while GATA4 showed additional immunoreactivity in testicular interstitial cells of XY gonadal primordia from 13.5 dpc staged embryos (Fig 2A). In developing ovaries (13.5 dpc), both proteins were co-expressed in the nuclei of somatic cells (Fig 2B). Only WT1 but no GATA4 was detectable in mesenchymal cells of the mesonephroi in both sexes (Fig 2A and 2B). This co-expression was observed throughout development into adulthood (data not shown). WT1 and GATA4 do not co-localize in cells of the mesonephros, which only contain WT1 protein. These data demonstrate that WT1 and GATA4 are co-expressed in Sertoli cells of seminiferous tubules of testes as well as in somatic cells of ovaries.

### *Vivo*-morpholino induced knockdown in *ex vivo* gonad cultures provides a tool to study gene function beyond the stage of sex determination

**Fig 3:**
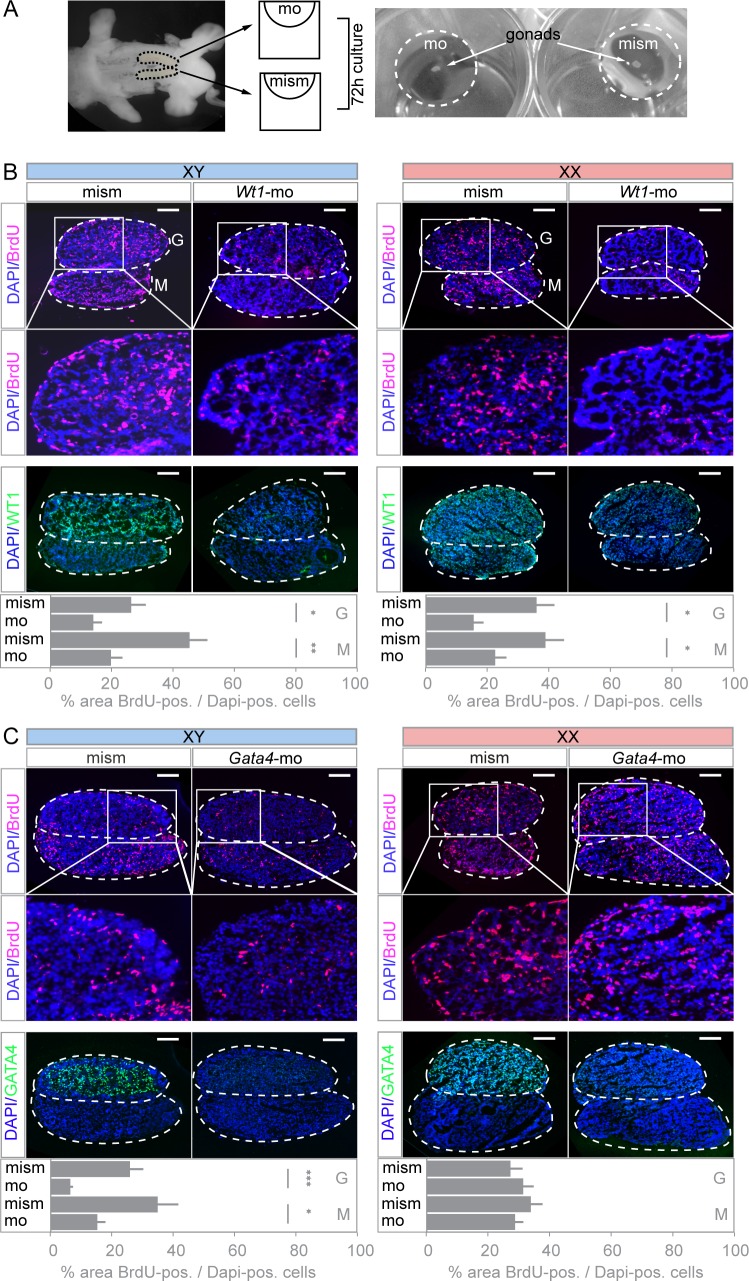
Experimental workflow and effect on cell proliferation upon WT1 or GATA4 knockdown. (A) Dissected XX and XY gonad/ mesonephroi regions (12.5 dpc) were incubated for 72 h in a droplet of medium supplemented with either *Wt1* or *Gata4 vivo*-morpholino. Transfection with the corresponding mismatch *vivo*-morpholinos was performed as control. Efficient gene silencing was assessed by immunostaining of WT1 (B) and GATA4 (C) in XX and XY gonad/ mesonephroi explants. Cell proliferation was determined by means of BrdU incorporation, and nuclei were visualized with Dapi. Proliferating cells were reduced in both XX and XY gonad/ mesonephroi explants upon WT1 knockdown (B), and knockdown of GATA4 only showed an effect on XY explants (C). BrdU-positive cells were counted in at least 5 tissue sections obtained from 3 different embryos and normalized to Dapi-stained nuclei. Scale bars indicate 100 μm. Error bars represent S.E.M., *p<0.05, **p<0.01, ***p<0.001, *t-*test. mo = *vivo*-morpholino. mism = *vivo*-morpholino-mismatch. G = gonad. M = mesonephros.

In order to study the functional relationship between WT1 and GATA4 past sex determination, we modified and combined an *ex vivo* droplet culture technique with an antisense strategy to knockdown WT1 and GATA4. The advantage of this approach is that it allows cultivating individual gonads, knocking-down both proteins in all expressing gonadal cell types, and assessing the same developmental stage in both sexes. The experimental set-up of the assay is shown in Fig 3A. Testes and ovaries were dissected from 12.5 dpc embryos and placed into a droplet of medium, supplemented with *vivo*-morpholino. The contra-lateral gonad served as a control and was treated with a mismatch-morpholino. The knockdown efficiency in individual gonads was assessed by immunohistochemistry using antibodies against either WT1 or GATA4. The observed homogenous decrease of WT1 and GATA4 proteins in XY and XX gonads suggests a high and reproducible delivery efficiency of the respective *vivo*-morpholinos (Fig 3B and 3C).

WT1 and GATA4 regulate cell proliferation in various tissues, and a reduced mitotic activity of progenitor cells contributes to the developmental defects in *Wt1*- and *Gata4*-deficient animals [32,33]. We therefore explored whether treatment with *Wt1* and *Gata4 vivo*-morpholinos was associated with impaired cell proliferation and whether gross morphological changes occurred upon the *vivo*-morpholino treatment. To address this, we visualized actively dividing cells of cultured gonadal primordia (12.5 dpc) by the means of BrdU incorporation (Fig 3B and 3C). Inhibition of *Wt1* significantly reduced the number of BrdU-positive cells in the gonads and mesonephroi of both sexes, whereas *Gata4 vivo*-morpholino treatment decreased cell proliferation only in tissues of males (Fig 3B and 3C). Thus, combining an *ex vivo* organ droplet culture technique with *vivo*-morpholinos provides an experimental tool that allows for an efficient and precisely timed knockdown of WT1 and GATA4 in embryonic testes and ovaries beyond the stage of sex determination.

### WT1 maintains sex-specific gene expression patterns beyond sex determination

Our analyses of *Wt1*^*-/-*^ embryonic gonads revealed abnormal expression of genes involved in sex determination and gonadal differentiation (Fig 1). Since WT1 regulates *Sry* expression, which is essential for transition of the bipotential gonad into a testis [8], changes in the gene expression pattern in male *Wt1*^*-/-*^ gonads could be a direct consequence of reduced SRY activity.

**Fig 4:**
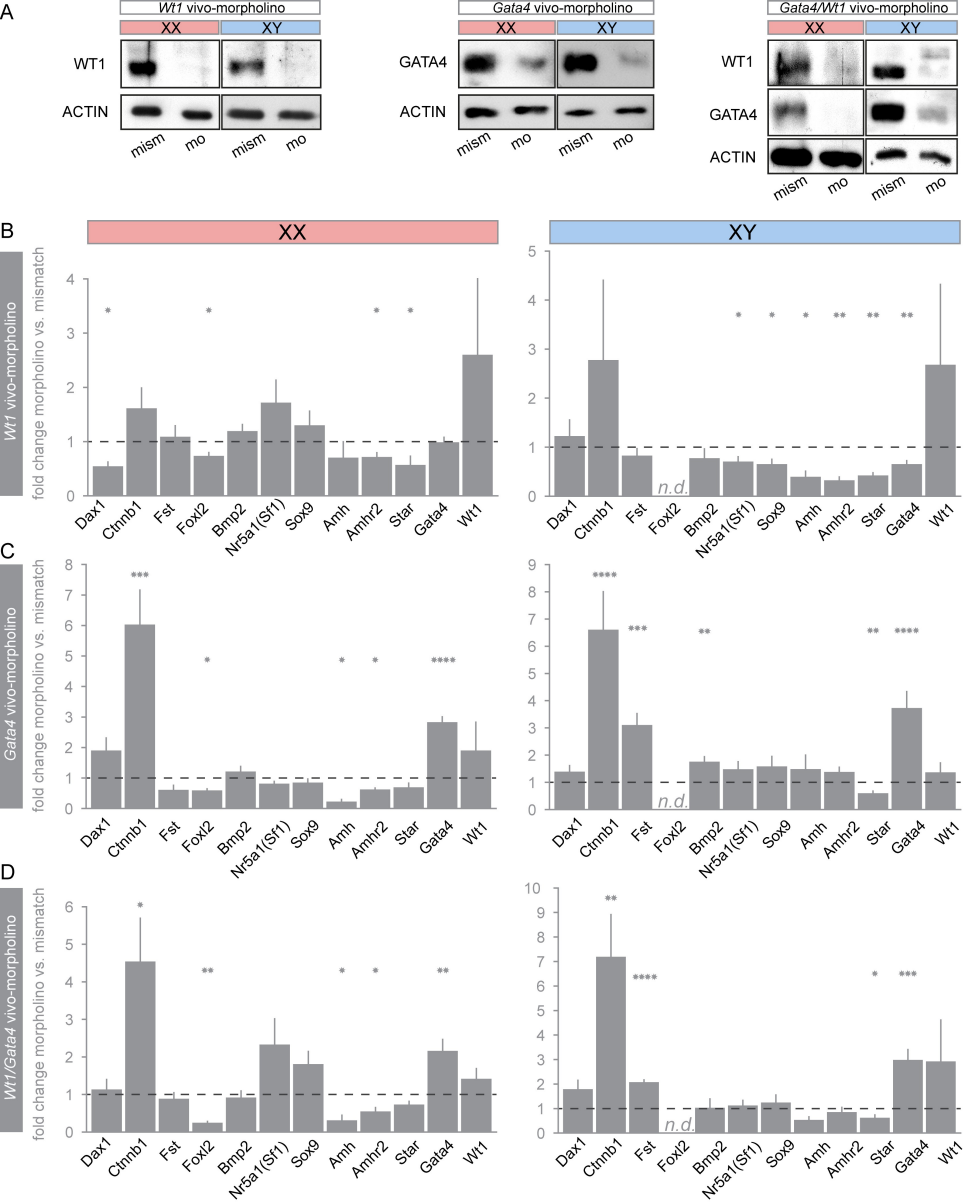
Single- and double-knockdown of WT1 and GATA4 in XX and XY gonads. (A-D) XX and XY gonads were dissected from murine embryos (12.5 dpc) and incubated for 72 h with *Wt1* and/ or *Gata4 vivo*-morpholinos. Transfection with appropriate mismatch *vivo*-morpholinos served as a negative control. For double-knockdown of WT1 and GATA4, *vivo*-morpholinos were applied simultaneously. (A) Efficient knockdown of WT1 and/or GATA4 was assessed by immunoblot analysis. (B-D) Relative transcript levels were determined by qRT-PCR and normalized to *Sdha* transcripts according to the 2^ΔΔCt^ method [34]. Results are shown as fold differences between cultures treated with mismatch *vs*. *Wt1-* (B) and mismatch *vs*. *Gata4-*morpholino (C), respectively. Panel D represents the effect of the double-knockdown of WT1 and GATA4. Error bars represent S.E.M. from independent biological replicates (n ≥ 5). scale = log2. Statistical differences are indicated by asterisks: *p<0.05, **p<0.01, ***p<0.001, ****p<0.0005, paired *t*-test. n.d., not detectable.

Still, the question arises whether WT1 is also necessary for maintaining a sex-specific gene expression pattern past the phase of sex determination, i.e. when SRY is normally down-regulated in developing murine testes (12.5 dpc). We applied the *ex vivo* droplet culture technique in order to address this issue. Knockdown efficiencies were assessed by immunoblot analyses using individual gonads of either sex (Fig 4A). Treatment with *Wt1 vivo*-morpholino significantly decreased *Dax1* and *Foxl2* mRNA levels in XX, but not in XY gonads (Fig 4B). As expected, *Foxl2* transcripts were not detectable in developing testes, in which *Nr5a1*, *Sox9*, *Amh*, and *Gata4* transcripts were significantly down-regulated in response to antisense inhibition of *Wt1*. This effect was not observed in XX gonads (Fig 4B). However, in both sexes, *Amhr2* and *Star* mRNA levels were significantly reduced upon WT1 knockdown, and this effect was more pronounced in XY than in XX gonadal primordia (Fig 4B). *Ctnnb1* and *Fst* transcript levels were not significantly altered between XY and XX gonads (Fig 4B). These data indicate that WT1 modulates the gonadal gene expression pattern in a sex-specific manner even beyond the stage of sex determination, i.e. after cessation of *Sry* expression.

### GATA4 is crucial for repression of ovarian promoting genes upon gonadal commitment to the male fate

Since WT1 synergizes with GATA4 in regulating genes involved in sex determination and gonadal differentiation [30], we used a specific antisense *vivo*-morpholino to assess the role of GATA4 in regulating gene expression downstream of WT1. Notably, knockdown of GATA4, either alone or combined with *Wt1* antisense treatment, did not change *Wt1* transcript levels (Fig 4C and 4D). Although GATA4 protein was clearly reduced by treatment with *Gata4 vivo*-morpholino (Fig 4A), *Gata4* mRNA levels were significantly increased in XY and XX gonadal explants (Fig 4C and 4D). Knockdown of GATA4 exerted specific effects on the gene expression pattern in developing XX *vs*. XY gonads, which were different from those observed upon *Wt1* silencing (Fig 4B and 4C). Whilst the knockdown of GATA4 reduced *Foxl2*, *Amh* and *Amhr2* transcripts in XX gonads, it decreased *Star* mRNA levels and, remarkably, increased ovarian promoting transcripts, i.e. *Ctnnb1*, *Fst*, and *Bmp2* transcripts in XY gonads (Fig 4C). Elevated *Ctnnb1* mRNA levels in response to *Gata4 vivo*-morpholino treatment were also observed in XX gonads (Fig 4C). Lastly, no significant changes in *Dax1*, *Nr5a1* and *Sox9* mRNA levels were seen (Fig 4C). These results indicate that GATA4 not only regulates the expression of *Foxl2* in XX gonads, but also represses ovarian promoting transcripts (*Fst*, *Bmp2*) in testes. Furthermore, GATA4 represses *Ctnnb1* and *Gata4* mRNAs in both, embryonic XY and XX gonads.

### WT1 requires GATA4 to propagate a sex-specific gene expression pattern

To further elucidate whether WT1 and GATA4 functionally cooperate during gonadal development, we applied both *vivo*-morpholinos at the same time for simultaneous translational inhibition of WT1 and GATA4. Immunoblot analysis confirmed the efficient down-regulation of both proteins in this combined approach (Fig 4A). The most striking effects of the simultaneous *Wt1/ Gata4 vivo*-morpholino treatment were observed on those transcripts that were predominantly down-regulated by sole *Wt1* inhibition (Fig 4B and 4D). In XY gonads, joint antisense treatment of *Wt1* and *Gata4* prevented the decrease of *Nr5a1*, *Sox9*, *Amh* and *Amhr2* transcript levels seen upon single *Wt1* inhibition (Fig 4B and 4D). In XX gonads, the same experimental approach abrogated the reduction of *Dax1* and *Star* mRNA levels caused by sole *Wt1* silencing (Fig 4D and 4B). In contrast, all analyzed genes that were modulated by single *Gata4* antisense treatment, were still regulated in the same manner by combined *Wt1/ Gata4* silencing, with the exception of the *Bmp2* and *Foxl2* transcripts. But, the latter of which showed a more pronounced effect, which is suggestive for the synergistic action of WT1 and GATA4 (Fig 4D). In summary, the double-knockdown approach indicates that WT1 requires GATA4 in order to maintain a sex-specific gene expression pattern and furthermore suggests that GATA4 fine-tunes correct thresholds of gene expression levels that are pivotal for the development of both, testes and ovaries.

### GATA4 represses the *Gata4* exon 1b isoform in testis

**Fig 5:**
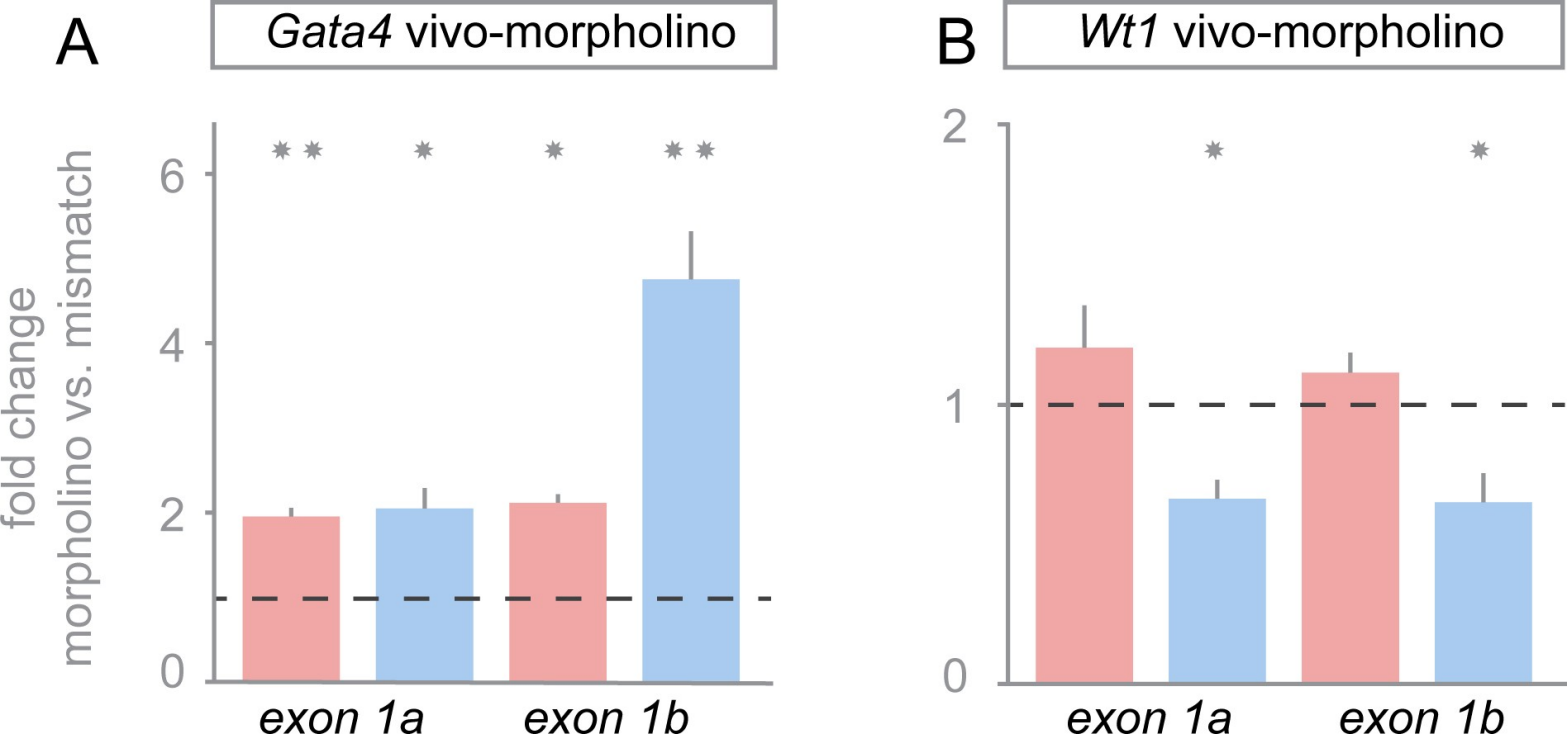
Sex-specific effect of WT1 and GATA4 knockdown on *Gata4 E1a* and *Gata4 E1b* isoforms expression. (A, B) Knockdown of GATA4 showed a strong significant increase in *Gata4 E1b* mRNA transcripts in XY but not in XX gonads. WT1 knockdown resulted in a significant decrease of *Gata4 E1b* and *Gata4 E1a* transcripts in XY gonads but not in XX gonads. Transcripts were measured by qRT-PCR and normalized to *Sdha* [34]. Results are shown as fold differences between cultures treated with mismatch *vs*. *Gata4-* (A) and mismatch *vs*. *Wt1-*morpholino (B). Error bars represent S.E.M. from independent biological replicates (n ≥ 5). Scale = log2. Statistical differences are indicated by asterisks: *p<0.05, **p<0.01, paired *t*-test.

Remarkably, despite a successful knockdown of the GATA4 protein, its transcript levels increased significantly (Fig 4A, 4C and 4D). This observation is in line with the previous report of a regulatory feedback loop in which GATA4 acts as a selective repressor of its own *Gata4 E1b* isoform. According to this model, GATA4 is normally transcribed from the *E1a* promoter only, while it represses its more distant *E1b* promoter [35]. However, when GATA4 levels decline due to reduced transcription of *Gata4 E1a*, the repressive effect is relieved and *Gata4* is transcribed from the *E1b* promoter. This mechanism ensures sufficient cellular GATA4 levels. Notably, *Gata4* deficient mice express elevated *Gata4 E1b* transcripts in their gonads [35]. Thus, we tested whether the same effect occurred in our experimental system upon *vivo*-morpholino knockdown of GATA4. Using *Gata4* isoform specific primers for qRT-PCR, *Gata4 E1b* mRNA levels were found to increase considerably upon GATA4 silencing in testes but not in ovaries (Fig 5A). On the contrary, *Gata4 E1b* and *Gata4 E1a* mRNA levels were both significantly decreased in XY gonads but remained unchanged in XX gonads upon *Wt1 vivo*-morpholino treatment (Fig 5B). Consistent with previous *in vivo* observations [35] these findings indicate that GATA4 down-regulates its own transcription from the *E1b* promoter in developing testes.

### WT1 and GATA4 affect the expression of hedgehog signaling molecules in developing XY and XX gonads

**Fig 6:**
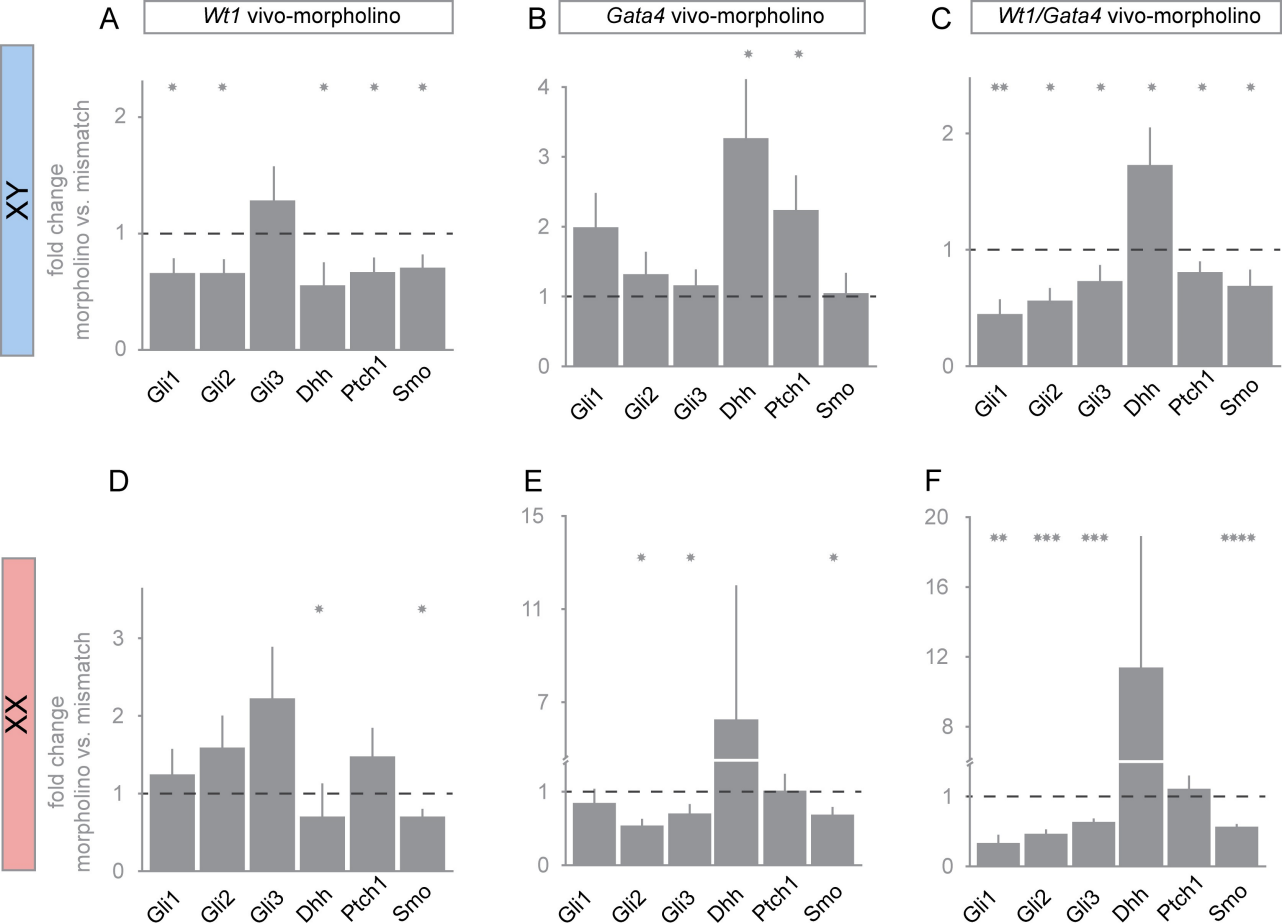
Single- and double-knockdown of WT1 and GATA4 affect mRNA levels of hedgehog pathway components in XY and XX gonads. XY (A-C) and XX (D-F) gonads, dissected from murine embryos (12.5 dpc), were incubated for 72 h with *Wt1* and/ or *Gata4 vivo*-morpholinos as described in Fig 4. Relative transcript levels were determined by qRT-PCR and normalized to *Sdha* transcripts according to the 2^ΔΔCT^ method [34]. Results are shown as fold differences between cultures treated with mismatch *vs*. *Wt1-* (A, D) and mismatch *vs*. *Gata4-*morpholino (B, E), respectively. Panels C and F represents the effect of double-knockdown of WT1 and GATA4. Error bars represent S.E.M. from independent biological replicates (n ≥ 8). scale = log2. Statistical differences are indicated by asterisks: *p<0.05, **p<0.01, ***p<0.005, ****p<0.001, paired *t*-test.

Recent findings highlight the role of the hedgehog (HH) signaling pathway in promoting fetal Leydig cell differentiation [36]. Sertoli cells express and secrete the ligand desert hedgehog (DHH), which binds to its receptor patched 1 (PTCH1) on cells that constitute the gonadal interstitium. Signaling through the HH pathway culminates in the activation of glioma-associated oncogene homolog transcription factors (GLI1, GLI2, GLI3) [37]. While the deletion of *Dhh* is associated with aberrant Leydig cell differentiation, the loss of either *Gli1* or *Gli2* does not affect their differentiation, suggesting redundant roles of the GLI factors [17,36,38]. WT1 has previously been shown to regulate the expression of *Dhh* and *Ptch1*, the latter being identified as a candidate WT1 target in the posterior taste field in the developing tongue [39,40]. However, whether other components of the HH signaling pathway are regulated in the gonads by either WT1 or GATA4 remains as yet unclear. In order to address this, we performed single and double *vivo*-morpholino knockdowns of WT1 and GATA4. In agreement with previous studies [41,42], *Dhh* transcripts were several orders of magnitude higher in developing XY gonads than in XX gonadal explants. Interestingly, the WT1 and GATA4 knockdowns exert opposing effects on gene expression of HH signaling molecules in XY gonads. Knockdown of WT1 reduced *Gli1*, *Gli2*, *Dhh*, *Ptch1* and *Smo* mRNA levels (Fig 6A), while *Gata4 vivo*-morpholino treatment significantly increased *Dhh* and *Ptch1* mRNA levels. Transcript levels of other assessed HH signaling molecules remained unchanged in the GATA4 knockdown (Fig 6B). Further, double-knockdown of WT1 and GATA4, significantly reduced transcript levels of all tested components of the HH signaling pathway in XY gonads, except for *Dhh* mRNA levels, which were significantly increased (Fig 6C). In XX gonads, mRNA levels of HH signaling molecules also changed upon the knockdown of WT1 and GATA4. While *Dhh* and *Smo* transcript levels were significantly reduced in developing ovaries in response to *Wt1* vivo-morpholino treatment (Fig 6D), knockdown of GATA4 caused a decrease of *Gli2*, *Gli3* and *Smo* mRNAs (Fig 6E). Double-knockdown of WT1 and GATA4 produced similar changes as single GATA4 inhibition in cultured XX gonads, and additionally lowered *Gli1* transcript levels (Fig 6F). In summary, these findings suggest that WT1 is necessary for the normal expression of components of the HH signaling pathway in the developing testis and ovary. As such, it contributes to correct differentiation of fetal Leydig cells in the XY gonad and to normal follicular development in the XX gonad. On the contrary, GATA4 inhibits *Dhh* and *Ptch* expression in the embryonic testis suggesting that GATA4 may be required to fine-tune their expression in order to acquire correct threshold levels.

## Discussion

The essential functions of WT1 and GATA4 during gonadal development are well documented, but it is poorly understood to what extent both transcription factors contribute to the sex differentiation process. We addressed this issue in a novel approach by combining an *ex vivo* organ culture technique with *vivo*-morpholino induced gene knockdown.

In order to establish readouts that represent sex-specific developmental gene expression patterns, we initially analyzed germline *Wt1* knockout mice at 13.5 dpc. The rationale for the sex-specific analysis was based on the fact that previous studies did not consider putative differences between males and females [43]. Consistent with the proposed role of WT1 in regulating *SRY* transcription [8,30], we observed the loss of the sexually dimorphic gonadal gene signature upon germline *Wt1* deletion (Fig 1). Considering that SRY, whose expression rapidly declines after 11.5 dpc, is a master regulator of male sex determination (reviewed in [44]), we investigated the importance of WT1 during gonadal differentiation shortly after sex determination and furthermore extended our analysis to the functional relationship with GATA4.

To analyze the functional interplay between WT1 and GATA4 in the expression of genes involved in sex determination and gonadal development, *ex vivo* cultures of murine embryonic gonads were combined with *vivo*-morpholino induced gene silencing. *Vivo*-morpholinos have been successfully used for the genomic characterization of WT1 targets in nephron progenitors [19] and, most recently, for identifying WT1 as a key regulator of fibroblast growth factor (FGF) signaling pathway genes in early kidney development [45]. Compared to *Wt1* and *Gata4* knockout mice, which exhibit abnormal morphology of their gonads and die at an early embryonic stage [3,12], the initial development of the gonadal ridges was preserved in our *ex vivo* preparations. Importantly, gene silencing with *vivo*-morpholinos is independent of the transcriptional network of factors influencing promoters/ enhancers that drive transgene expression in conditionally mutant mice. For example, due to the differential expression of *Amh* in both males and females, the use of an *Amh-Cre* deleter mouse strain enables gene recombination only in the testis, while *vivo*-morpholino treatment was equally effective in down-regulating WT1 and GATA4 in both sexes (Figs 3B, 3C and 4). Another advantage of this approach is that the initiation of *Wt1* and *Gata4* gene silencing at the same developmental stage in XY and XX gonads allows for a direct comparison between sex-specific effects of both proteins. Furthermore, simultaneous inactivation of two or several genes can easily be accomplished in *ex vivo* cultured tissues, thus offering the opportunity to study redundant and/or combined effects of transcription factors.

Consistent with previous *in vivo* findings [33,39], morpholino-induced knockdown indicates that reduced cell proliferation contributes to abnormal morphogenesis of the gonads upon WT1 and GATA4 depletion (Fig 3B and 3C). Since GATA4 reduces BrdU incorporation only in developing testes but not in ovaries (Fig 3C), non-specific adverse effects of the *vivo*-morpholinos are unlikely to account for the changes in cell proliferation in XX gonads. With regard to the interpretation of data one should be aware that *ex vivo* cultured gonads may exhibit a developmental delay by approximately 24 h in comparison with gonads *in vivo* [46]. Due to the limited viability of the *ex vivo* preparations, later stages of gonadal development were not studied herein. Nevertheless, this can be done in future investigations by shifting the timeframe for tissue dissection and culture towards a later time point.

The observed down-regulation of *Nr5a1*, *Sox9* and *Amh* only in XY but not in XX gonads indicates that WT1 is indeed necessary for maintaining a sex-specific gene signature in the developing testis (Fig 4B). *Wt1 vivo*-morpholino treatment, initiated at the same developmental stage also revealed specific effects of WT1 in embryonic ovaries as exemplified by the reduction of *Dax1* and *Foxl2* mRNA levels (Fig 4B). It has recently been reported that FOXL2 antagonizes the stimulatory effect of WT1(-KTS) on *Nr5a1* expression by interacting with the proximal *Nr5a1* promoter region [47]. Our data provide evidence that WT1 also interferes with the transcriptional network controlling ovarian development. FOXL2 is expressed in somatic cells of XX gonads [48], and *Foxl2*-deficient mice lack primary follicles in their disorganized ovaries [48]. By sustaining normal *Foxl2* expression in XX gonads (Figs 1A and 4B) WT1 up-regulates its own competitor on testis promoting target genes [47]. Hence, WT1 normally activates *Nr5a1* expression in somatic cells of the testis (Figs 1A and 4B) [6], while FOXL2—up-regulated by WT1—inhibits *Nr5a1* expression in the developing ovary. A similar sex-characteristic regulatory mechanism may also apply to the *Sox9* gene whose expression is inhibited by FOXL2 [49]. Notably, *Foxl2* transcripts were also significantly reduced by antisense inhibition of GATA4 in developing ovaries (Fig 4C) as previously reported in *Sf1-Cre;Gata4*^*flx/flx*^ mice [33].

Analysis of single *Gata4 vivo*-morpholino treated gonads confirmed the regulation of *Star* [50] and led to the identification of novel downstream genes such as the ovarian markers *Ctnnb1*, *Fst*, and *Bmp2*. Our data suggest that GATA4 does not contribute to the sexually dimorphic expression of *Ctnnb1* (Fig 4C and 4D), but rather sustains basal levels of β-catenin necessary for gonadal development in both, males and females [51]. FST is a secreted glycoprotein, which prevents the formation of testis-specific vasculature and germ cell death through inhibition of activin B [52,53]. Therefore, GATA4 may facilitate testis formation by inhibiting *Fst* expression, which in turn leads to increased activin B levels. This interpretation is in line with the observed increase in *Bmp2* expression in testis upon GATA4 knockdown, because in ovaries FOXL2 and BMP2 cooperatively enhance *Fst* expression [54]. This observation suggests that GATA4 is required in order to repress ovarian promoting transcripts when cell fate decisions follow along the male pathway (Fig 4C).

Activation of the *Amh* promoter by GATA4 has been reported earlier [55], but the specific decrease of *Amh* and *Amhr2* mRNA levels in embryonic ovaries upon GATA4 antisense inhibition is on the first view unexpected (Fig 4C and 4D). Given that *Amh* expression in the ovary generally is very low (Fig 1B), activation of *Amh* and *Amhr2* by GATA4 may have no physiological significance.

Regulatory feedback mechanisms could be detected on the level of *Gata4* itself. Despite efficient inhibition of GATA4 protein translation (Figs 3C and 4A), its mRNA levels were significantly increased after 72 h of *Gata4 vivo*-morpholino treatment (Fig 4C and 4D). Such a robust GATA4 feedback regulation has been also described in other models [56]. In the gonads, this feedback control may involve the generation of two major alternative *Gata4* transcripts, which differ in their first exon [35]. While the proximal *Gata4 E1a* RNA predominates in adult tissues, the developing gonads express both, the *Gata4 E1a* and the *E1b* transcripts at similar high levels [35]. Notably, the translation start site is located in exon2 of the *Gata4* gene, and alternative exon1a and 1b are both non-coding. In line with repression of the *Gata4 E1b* promoter by GATA4 in Leydig and Sertoli cells, elevated *Gata4 E1b* transcripts have recently been reported in *Gata4* null gonads [35], which is supported by our data (Figs 4C, 4D and 5). Our findings further suggest that activation of transcription from the *Gata4 E1b* promoter provides an important back-up mechanism to ensure sufficient GATA4 levels for repressing ovarian promoting genes in testes. Despite elevated *Gata4* exon1b mRNA levels GATA4 protein was reduced in the *Gata4 vivo*-morpholino treated cultures (Fig 4A) due to inhibition of protein translation.

GATA4 has been found to physically interact with WT1 in the co-regulation of the promoters of the *Amh* and *Sry* genes, respectively [30]. This was our rationale for investigating the combined effects of WT1 and GATA4 on gonadal genes by joint antisense inhibition of both molecules. For most genes (*Dax1*, *Nr5a1*, *Sox9*, *Amh*, *Amhr2*), combined down-regulation of GATA4 prevented the decrease of mRNAs that was caused by sole *Wt1 vivo*-morpholino treatment. This indicates that WT1 requires GATA4 in order to establish and maintain a sex-specific gene expression signature in the developing gonads. In addition, simultaneous knockdown of WT1 and GATA4 suggests a synergistic stimulatory effect of both proteins on the expression of *Foxl2* (Fig 4B and 4D). Similar changes in transcript levels were caused by combined WT1 and GATA4 knockdown and by sole GATA4 depletion (Fig 4C and 4D).

Recent findings indicate that the hedgehog signaling pathway is important for male sex differentiation. Desert hedgehog (DHH) is a secreted protein expressed in Sertoli cells of the embryonic testis that induces precursor cells surrounding the embryonic testis cords to become androgen producing Leydig cells [36,57]. Male *Dhh* null mice are infertile lacking development of mature sperm cells likely due to impaired differentiation of peritubular myoid cells in the absence of HH signaling [58–60]. Down-regulation of *Dhh* transcripts in *Wt1 vivo*-morpholino treated XY gonadal explants (Fig 6A) is in line with a previous report of reduced *Dhh* expression in *Wt1*-deficient testes of adult mice [39]. Furthermore, our findings corroborate a most recent study illustrating decreased *Dhh* mRNA levels in *Wt1*-deficient testes at E13.5 [61]. By demonstrating that WT1 knockdown also decreased *Gli1*, *Gli2*, *Ptch1* and *Smo* transcripts in embryonic XY gonads (Fig 6A), our findings suggest that WT1 activates the HH pathway at multiple stages. Notably, *Wt1 vivo*-morpholino treatment did not significantly change transcript levels of *Gli3*, which is thought to function mainly as a transcriptional repressor in the absence of active HH signals [62]. Our data further suggest that–opposite to WT1 –GATA4 inhibits *Dhh* and *Ptch1* expression in XY gonads (Fig 6B). Transcript levels of *Dhh* were significantly lower in XX than in XY gonads, suggesting a higher activity of the HH signaling pathway in embryonic testes than in embryonic ovaries [41,42]. Yet, given the observation that activation of the HH pathway in the mouse fetal ovary leads to the ectopic appearance of fetal Leydig cells and female pseudohermaphroditism [63], abnormal expression of WT1 and/ or GATA4 may impair gonad development secondary to changes in HH signaling.

Targeted *Wt1* inactivation in Sertoli cells has recently been reported to impair the differentiation of peritubular myoid cells and fetal Leydig cells during testis development [64,65] even though WT1 is normally absent from both, androgen producing Leydig cells in fetal testes and theca precursor cells in fetal ovaries. Disturbed Leydig cell development in response to *Wt1* inactivation in Sertoli cells is therefore likely an indirect effect, e.g. resulting from the loss of Sertoli cell-derived signals that promote Leydig cell differentiation. Notably, Leydig cells and theca-interstitial cells are the steroidogenic cells and the major sites of *Nr5a1* and *Star* expression in male and female gonads, respectively. We therefore assume that the changes in *Nr5a1* and *Star* expression caused by WT1 knockdown are also indirect, i.e. due to abnormal paracrine signaling from Sertoli and granulosa cells to interstitial Leydig and theca precursor cells, respectively (Fig 4).

**Fig 7:**
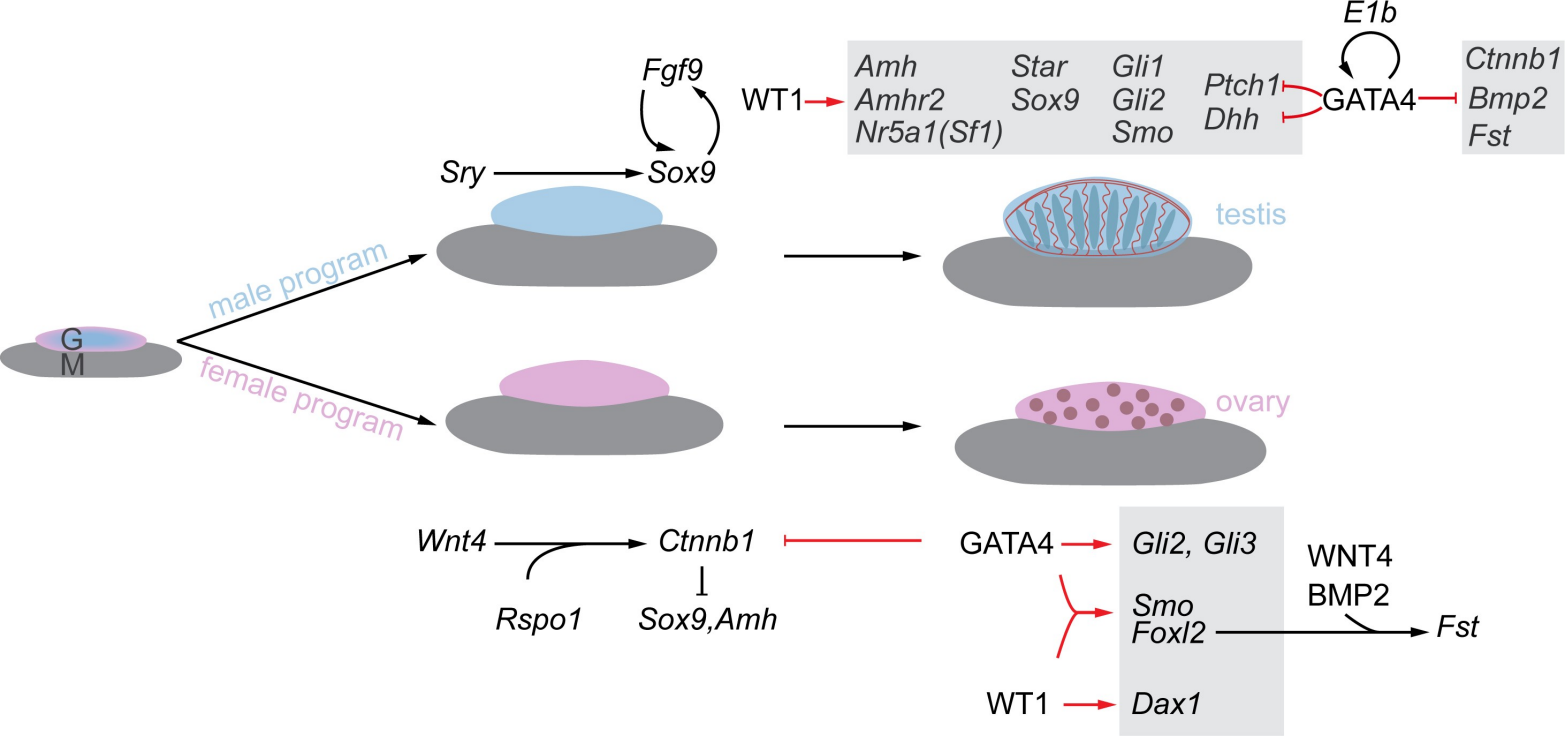
WT1 and GATA4 in the regulation of sex-specific gonadal gene expression. During testis development, WT1 is required for activating various testis promoting genes, i.e. *Amh*, *Amhr2*, *Nr5a1*(*Sf1)*, *Star*, *Sox9*, *Gli1*, *Gli2*, *Smo*, *Dhh and Ptch1*. GATA4 represses ovarian promoting transcripts such as *Ctnnb1*, *Bmp2*, and *Fst*, in the testis, and thereby may be essential for the maintenance of the testis phenotype. GATA4 is also involved in repressing *Dhh* and *Ptch1* transcript levels in embryonic testis, which may contribute to the fine-tuning of their threshold levels. Given such a pivotal role of GATA4, it is likely that sufficient GATA4 levels are ensured by a back-up mechanism provided by the GATA4 E1b isoform in the testis. During ovary development, i.e. in the absence of SRY, WNT4 and RSPO-1 levels are stabilized and promote canonical Wnt signaling. In the ovary, WT1 is required for *Dax1* expression. GATA4 acts as a repressor of *Ctnnb1* and serves as an activator for the transcripts *Gli2*, *Gli3*, and *Smo*, the latter of which may be regulated synergistically by both WT1 and GATA4. WT1 and GATA4 may also synergize on *Foxl2* transcription, which contributes to an ovarian specific signature. Suggested stimulatory (→) and inhibitory (⊥) pathways identified herein are marked in red color.

Although it remains to be determined which of the observed changes in gonadal gene expression are immediate consequences of WT1 and GATA4 protein depletion, our findings nevertheless contribute to the overall understanding of gonad development and can be summarized as represented in Fig 7. WT1 is required to establish a sex-specific gene expression program in developing gonads. Furthermore, it exerts a crucial role beyond the stage of sex determination by maintaining the expression of *Nr5a1*, *Sox9*, *Amh*, *Star* and *Amhr2*, in testis, as well as *Dax1* and *Foxl2* in ovaries. These effects of WT1 on gonadal gene expression are strictly dependent on normal expression of GATA4, which represses ovarian promoting transcripts in XY gonads. Hence, down-regulation of GATA4 in embryonic gonads will not only disrupt the sex-specific gene expression signature initiated and maintained by WT1, but also increase ovarian promoting transcripts in testis. This may eventually give rise to abnormal development of the XY gonads. Thus, GATA4 fulfills a dual role as a transcription factor by repressing ovarian promoting transcripts in embryonic testes and by enhancing the expression of ovarian promoting genes, e.g. *Foxl2* in developing ovaries.
